# Gene Expression Analysis in Human Breast Cancer Associated Blood Vessels

**DOI:** 10.1371/journal.pone.0044294

**Published:** 2012-10-02

**Authors:** Dylan T. Jones, Tanguy Lechertier, Richard Mitter, John M. J. Herbert, Roy Bicknell, J. Louise Jones, Ji-Liang Li, Francesca Buffa, Adrian L. Harris, Kairbaan Hodivala-Dilke

**Affiliations:** 1 Centre for Tumour Biology, Barts Cancer Institute, Queen Mary University of London, London, United Kingdom; 2 Bioinformatics and Biostatistics Service, Cancer Research United Kingdom, London, United Kingdom; 3 Immunity and Infection, College of Medical and Dental Sciences, University of Birmingham, Birmingham, United Kingdom; 4 Molecular Oncology, The Weatherall Institute of Molecular Medicine, University of Oxford, Oxford, United Kingdom; Children's Hospital Boston & Harvard Medical School, United States of America

## Abstract

Angiogenesis is essential for solid tumour growth, whilst the molecular profiles of tumour blood vessels have been reported to be different between cancer types. Although presently available anti-angiogenic strategies are providing some promise for the treatment of some cancers it is perhaps not surprisingly that, none of the anti-angiogenic agents available work on all tumours. Thus, the discovery of novel anti-angiogenic targets, relevant to individual cancer types, is required. Using Affymetrix microarray analysis of laser-captured, CD31-positive blood vessels we have identified 63 genes that are upregulated significantly (5–72 fold) in angiogenic blood vessels associated with human invasive ductal carcinoma (IDC) of the breast as compared with blood vessels in normal human breast. We tested the angiogenic capacity of a subset of these genes. Genes were selected based on either their known cellular functions, their enriched expression in endothelial cells and/or their sensitivity to anti-VEGF treatment; all features implicating their involvement in angiogenesis. For example, *RRM2*, a ribonucleotide reductase involved in DNA synthesis, was upregulated 32-fold in IDC-associated blood vessels; *ATF1*, a nuclear activating transcription factor involved in cellular growth and survival was upregulated 23-fold in IDC-associated blood vessels and *HEX-B*, a hexosaminidase involved in the breakdown of GM2 gangliosides, was upregulated 8-fold in IDC-associated blood vessels. Furthermore, *in silico* analysis confirmed that *AFT1* and *HEX-B* also were enriched in endothelial cells when compared with non-endothelial cells. None of these genes have been reported previously to be involved in neovascularisation. However, our data establish that siRNA depletion of *Rrm2, Atf1* or *Hex-B* had significant anti-angiogenic effects in VEGF-stimulated *ex vivo* mouse aortic ring assays. Overall, our results provide proof-of-principle that our approach can identify a cohort of potentially novel anti-angiogenic targets that are likley to be, but not exclusivley, relevant to breast cancer.

## Introduction

Angiogenesis, the formation of new blood vessels from pre-existing vasculature, is critical for tumour growth and cancer progression, implying that anti-angiogenic drugs are likely to be of importance in the treatment of neoplasia [1], [2]. Angiogenesis is influenced by several growth factors, such as vascular endothelial growth factor (VEGF) and basic fibroblast growth factor (bFGF) [3], [4]. Indeed, anti-angiogenic strategies targeting VEGF have shown some considerable promise, but improvements are still needed. Identifying gene expression changes between tumour-associated blood vessels and those in normal tissues may provide us with new anti-angiogenic targets. Some data have suggested that blood vessels supplying tumours express genes not expressed in blood vessels in normal tissues [5]–[9]. Although results from such studies have yet to be verified, given that the molecular ‘zipcodes’ of tumour-associated vasculatures may be different between cancer types, identifying anti-angiogenic targets relevant to tumour types may have significant benefits over currently available strategies [10]–[14].

Tumours consist of a mixture of cancer and stromal compartments, which have their own gene expression profiles and, as such, analysis of whole tumours is not necessarily appropriate when designing anti-angiogenic agents [15]–[24]. In addition cell culture based studies are open to the criticism that they induce molecular changes, making results less relevant to the disease in the whole organism [7], [8]. An alternative method is to use laser capture microdissection (LCM), which allows for the isolation of specific tissues or cells directly from whole tissue sections [5], [9], [25]–[28]. LCM has been used successfully for PCR- and microarray analysis of specific cell populations including blood vessels [5], [9], [25], [27], [29]. CD31 (PECAM1) is known to be a suitable marker for the identification of angiogenic blood vessels in many tissues, including breast cancer and is used as such in the pathological analysis of breast cancer [30], [31].

**Figure 1:**
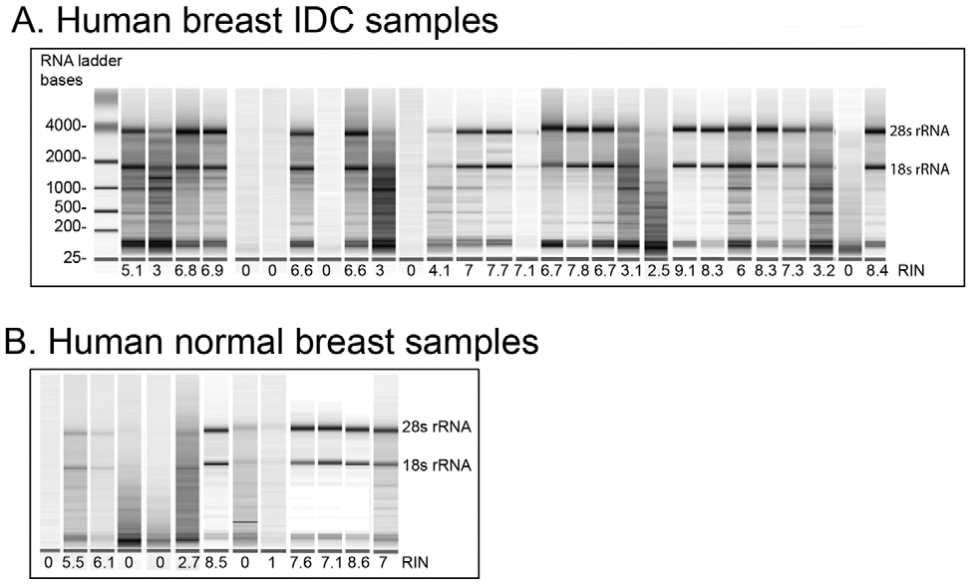
RNA profile and quality of frozen breast IDC samples. RNA profiles of (**A**) IDC and (**B**) normal breast samples using Agilent bioanalyser. RNA bands correspond to 28S and 18S rRNA. RNA quality was rated according to the RNA integrity number (RIN).

Here we have developed a method for the detection of CD31 in human breast cancer and normal human breast, followed by LCM of CD31-positive blood vessels and subsequent expression array analysis. We have identified 7 downregulated and 63 upregulated genes associated with human breast cancer CD31-postive blood vessels. Our data has demonstrated that at least 3 of these genes, *Rrm2, Atf1* and *Hex-B*, when depleted in mouse aortic rings, have anti-angiogenic effects, validating our approach to the discovery of new anti-angiogenic targets.

**Figure 2:**
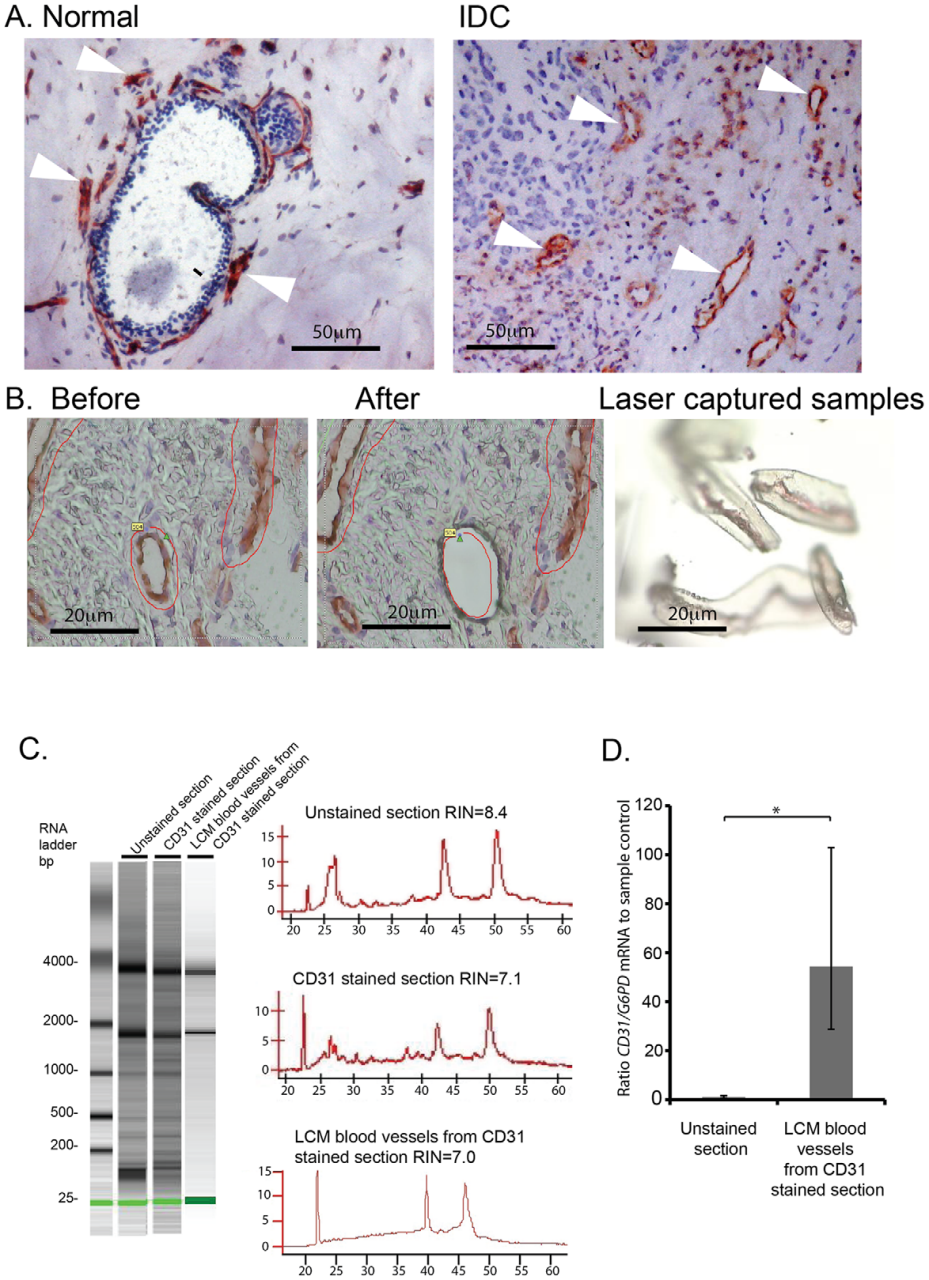
Combined CD31-immunostaining and LCM of blood vessels in breast tissue. (**A**) Normal and IDC breast sections were stained with anti-CD31 monoclonal antibody using our modified staining protocol. Blood vessels are stained in red with CD31-AEC (*Arrowheads*, blood vessels), and cell nuclei are stained in blue with Cresyl Violet. (**B**) PALM laser microdissection of CD31-positive blood vessels. (**C**) Agilent bioanalyser of RNA from LCM blood vessels compared with unstained control and CD31 stained sections. RNA quality was rated according to the RNA integrity number (RIN). Histograms correspond to RNA bioanalyser profiles. (**D**) Real-time PCR of CD31 mRNA expression in LCM blood vessels from breast tissue confirms high level of CD31 in laser captured material. *CD31* mRNA expression was given as a ratio to *G6PD* mRNA (internal control), and the data represented relative to unstained sample control (n = 2, ± fold range, *p<0.05).

## Methods

### Ethics Statement

Frozen human breast tissues were obtained from Barts and The London School of Medicine and Dentistry, Queen Mary University and Oxford John Radcliffe Hospital Biobank, a Human Tissue Authority Licensed Research Tissue Bank (HTA License number 12217). All patients gave informed written consent. For samples from Queen Mary University, the project was covered by ethics reference 05/Q0403/199 from the North East London ethics committee. For samples from Oxford John Radcliffe Hospital Biobank, the project was covered by ethics reference 09/H0606/5 from the National Research ethics service Oxfordshire. All animals were used in accordance with UK Home Office regulations and approved by the Queen Mary University of London and Oxford University ethics committee.

**Figure 3:**
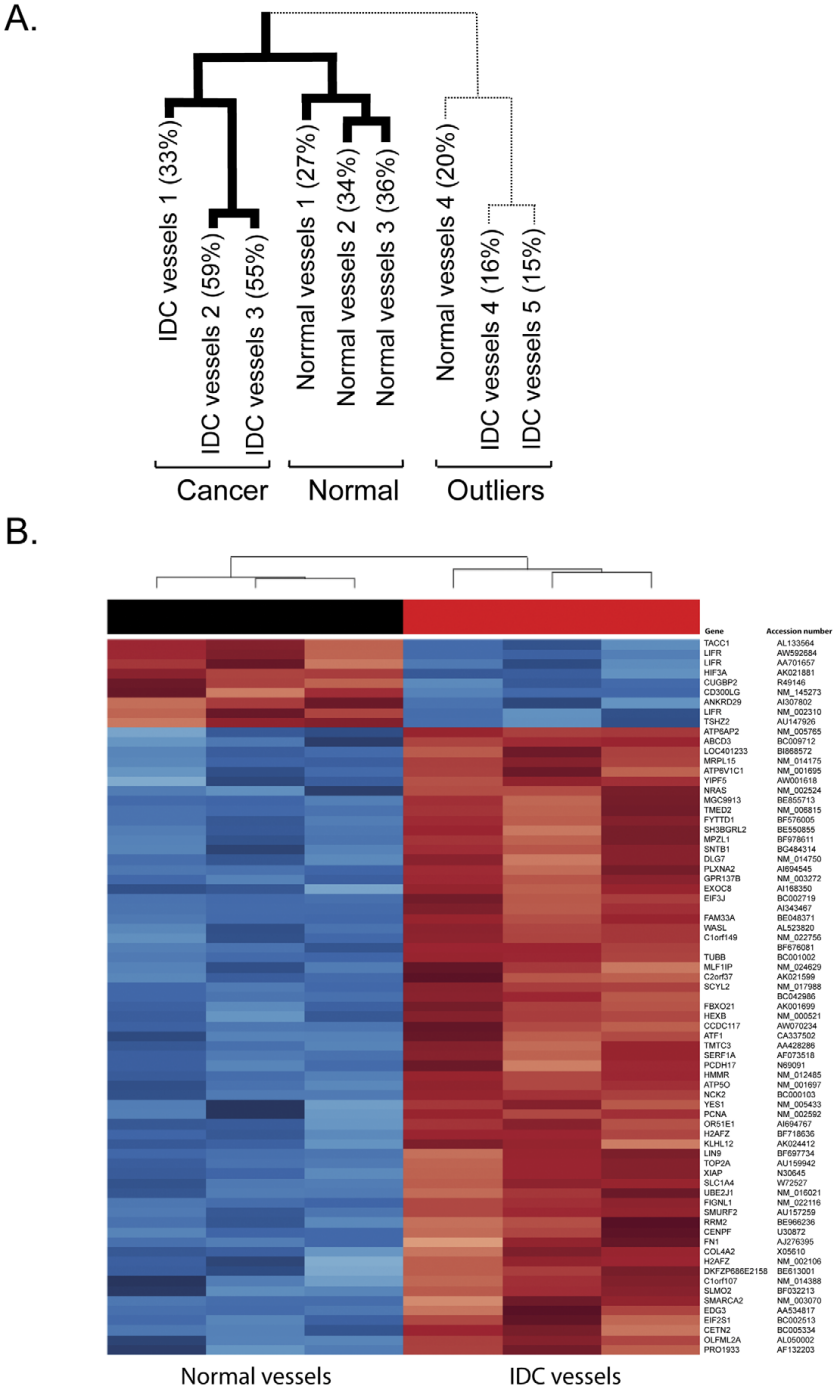
Affymetrix data from LCM blood vessels. (**A**) Hierarchal clustering of laser captured blood vessels from 4 normal and 5 IDC samples with percentage present call rate. (**B**) Heat map that shows the trend in expression of 73 probe-sets, 70 genes across the six samples. The blue indicates under-expression while the red indicates over-expression with gene name and accession number given.

**Table 1:** Seven of the most downregulated genes in breast IDC associated blood vessels with q<0.05.

Gene	Accession	Description	Fold change
***TACC1***	AL133564	Transforming, acidic coiled-coil containing protein 1	0.09
***CUGBP2***	R49146	CUG triplet repeat, RNA binding protein 2	0.08
***TSHZ2***	AU147926	Teashirt zinc finger homeobox 2	0.07
***HIF3A***	AK021881	Hypoxia inducible factor 3, alpha subunit	0.05
***LIFR***	NM_002310	Leukemia inhibitory factor receptor alpha	0.04
***ANKRD29***	AI307802	Ankyrin repeat domain 29	0.04
***CD300LG***	NM_145273	CD300 molecule-like family member g	0.01

**Table 2:** Sixty-three of the most upregulated genes in breast IDC associated blood vessels with greater than 5 fold change and q<0.05.

Gene	Accession	Description	Fold change
	AI343467	*NA*	72
	BC042986	*NA*	65
***C2orf37***	AK021599	*Chromosome 2 open reading frame 37*	64
***H2AFZ***	NM_002106	*H2A histone family, member Z*	40
***TOP2A***	AU159942	*Topoisomerase (DNA) II alpha 170kDa*	39
***EDG3***	AA534817	*Endothelial differentiation, sphingolipid G-protein-coupled receptor, 3*	37
***PCNA***	NM_002592	*Proliferating cell nuclear antigen*	32
***RRM2***	BE966236	*Ribonucleotide reductase M2 polypeptide*	32
***FN1***	AJ276395	*Fibronectin 1*	31
***CENPF***	U30872	*centromere protein F, 350/400ka (mitosin)*	25
***TMTC3***	AA428286	*Transmembrane and tetratricopeptide repeat containing 3*	25
***MRPL15***	NM_014175	*Mitochondrial ribosomal protein L15*	24
***ATF1***	CA337502	*Activating transcription factor 1*	23
***NRAS***	NM_002524	*Neuroblastoma RAS viral (v-ras) oncogene homolog*	22
***PCDH17***	N69091	*Protocadherin 17*	22
***C1orf107***	NM_014388	*Chromosome 1 open reading frame 107*	21
***PRO1933***	AF132203	*PRO1933*	21
***MLF1IP***	NM_024629	*MLF1 interacting protein*	20
***TMED2***	NM_006815	*Transmembrane emp24 domain trafficking protein 2*	20
***ATP5O***	NM_001697	*ATP synthase, H+ transporting, mitochondrial F1 complex, O subunit*	19
***XIAP***	N30645	*X-linked inhibitor of apoptosis*	19
***ABCD3***	BC009712	*ATP-binding cassette, sub-family D (ALD), member 3*	17
***TUBB***	BC001002	*Tubulin, beta*	17
***SLMO2***	BF032213	*Slowmo homolog 2 (Drosophila)*	17
***YES1***	NM_005433	*v-yes-1 Yamaguchi sarcoma viral oncogene homolog 1*	17
***SH3BGRL2***	BE550855	*SH3 domain binding glutamic acid-rich protein like 2*	17
***ATP6AP2***	NM_005765	*ATPase, H+ transporting, lysosomal accessory protein 2*	16
***MGC9913***	BE855713	*Hypothetical protein MGC9913*	15
***MPZL1***	BF978611	*Myelin protein zero-like 1*	15
***DKFZP686E2158***	BE613001	*Hypothetical protein LOC643155*	15
***EIF2S1***	BC002513	*Eukaryotic translation initiation factor 2, subunit 1 alpha*	15
***SERF1A***	AF073518	*Small EDRK-rich factor 1A (telomeric)*	14
***CETN2***	BC005334	*Centrin, EF-hand protein, 2*	13
***DLG7***	NM_014750	*Discs, large homolog 7 (Drosophila)*	13
***SLC1A4***	W72527	*Solute carrier family 1 (glutamate/neutral amino acid transporter), member 4*	13
***SMARCA2***	NM_003070	*SWI/SNF related, matrix associated, actin dependent regulator of chromatin, subfamily a, member 2*	13
***ATP6V1C1***	NM_001695	*ATPase, H+ transporting, lysosomal 42kDa, V1 subunit C1*	13
***GPR137B***	NM_003272	*G protein-coupled receptor 137B*	13
***C1orf149***	NM_022756	*Chromosome 1 open reading frame 149*	12
***UBE2J1***	NM_016021	*Ubiquitin-conjugating enzyme E2, J1*	12
***CCDC117***	AW070234	*Coiled-coil domain containing 117*	12
***KLHL12***	AK024412	*Kelch-like 12 (Drosophila)*	11
***EXOC8***	AI168350	*Exocyst complex component 8*	11
***GPR164***	AI694767	*Olfactory receptor, family 51, subfamily E, member 1*	11
***COL4A2***	X05610	*Collagen, type IV, alpha 2*	10
***YIPF5***	AW001618	*Yip1 domain family, member 5*	10
***EIF3J***	BC002719	*Eukaryotic translation initiation factor 3, subunit J*	9
***FYTTD1***	BF576005	*Forty-two-three domain containing 1*	9
***SNTB1***	BG484314	*Syntrophin, beta 1 (dystrophin-associated protein A1, 59kDa, basic component 1)*	9
***LOC401233***	BI868572	*Similar to HIV TAT specific factor 1; cofactor required for Tat activation of HIV-1 transcription*	9
***SMURF2***	AU157259	*SMAD specific E3 ubiquitin protein ligase 2*	8
***HEXB***	NM_000521	*Hexosaminidase B (beta polypeptide)*	8
***NCK2***	BC000103	*NCK adaptor protein 2*	8
	BF676081	*NA*	7
***SCYL2***	NM_017988	*SCY1-like 2 (S. cerevisiae)*	7
***OLFML2A***	AL050002	*Olfactomedin-like 2A*	7
***LIN9***	BF697734	*Lin-9 homolog (C. elegans)*	7
***WASL***	AL523820	*Wiskott-Aldrich syndrome-like*	6
***PLXNA2***	AI694545	*Plexin A2*	6
***FBXO21***	AK001699	*F-box protein 21*	6
***HMMR***	NM_012485	*Hyaluronan-mediated motility receptor (RHAMM)*	6
***FAM33A***	BE048371	*Family with sequence similarity 33, member A*	5
***FIGNL1***	NM_022116	*Fidgetin-like 1*	5

**Table 3:** Molecular and Cellular Function Ingenuity analysis of differentially regulated genes in human breast IDC associated blood vessels.

Name	p-value	Molecules
Cellular Assembly and Organisation	3.63E-07	11
Cellular Function and Maintenance	2.63E-05	9
Protein Synthesis	3.85E-05	8
Cell Cycle	2.12E-04	7
Energy Production	2.71E-04	3

**Table 4:** Canonical Ingenuity pathway analysis of differentially regulated genes in human breast IDC associated blood vessels.

Pathway	p-value	Molecules
Role of BRCA1 in DNA Damage Response	0.008	2
Ephrin Receptor Signalling	0.011	3
Protein Ubiquitination Pathway	0.013	3
Integrin Signalling	0.015	3
Axonal Guidance Signalling	0.017	4
BMP Signalling Pathway	0.018	2
TGF-β Signalling	0.019	2
VEGF Signalling	0.021	2

### Breast tissue, preparation and processing

Twenty-eight breast IDC and 13 normal breast samples were screened for suitability. Eight μm sections of tissue from frozen breast blocks were first trimmed using a cryostat set at −30°C to test RNA quality and morphology using haematoxylin staining. Only samples with RNA integrity number (RIN) of 6 or more were used (see RNA extraction protocol). RNase-free technique was used throughout the procedure using RNase-ZAP (Ambion). Up to 3 sections 8 µm thick were cut with the cryostat and placed on a treated PALM membrane polyethylene naphthalate slides (PALM Microlaser Technologies, Bernried, Germany). Up to 20 slides were collected per patient. Slides were stored on dry ice until sectioning was finished then immediately transferred to a −80°C freezer for long-term storage. The slides were stored up to 2 months before LCM.

**Figure 4:**
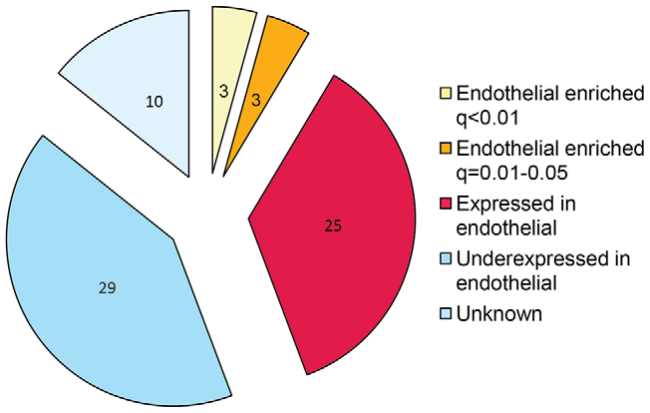
Pie chart of differentially expressed genes in breast IDC LCM blood vessels cross-referenced to endothelial cell libraries. *In-silico* subtraction, employing maximum likelihood statistics, between endothelial and non-endothelial pools found endothelial enriched genes and were used to prioritise LCM candidate genes. To account for multiple testing errors, a FDR was employed to calculate the significance of the genes being endothelial specific (q<0.05).

**Table 5:** List of genes enriched in endothelial cells in LCM samples.

Gene	Accession	Endothelial EST log2 fold change	q-value
***SMURF2***	**AU157259**	**9.00**	**<0.01**
***LIFR***	**NM_002310**	**8.00**	**<0.01**
***HEXB***	**NM_000521**	**3.36**	**<0.01**
***C1orf107***	**NM_014388**	**4.46**	**0.01**
***ATF1***	**CA337502**	**3.00**	**0.02**
***NRAS***	**NM_002524**	**4.13**	**0.04**
*TACC1*	AL133564	2.40	0.09
*GPR137B*	NM_003272	2.00	0.1
*YIPF5*	AW001618	1.77	0.17
*YES1*	NM_005433	2.55	0.18
*ATP5O*	NM_001697	1.08	0.26
*TMED2*	NM_006815	1.13	0.29
*EXOC8*	AI168350	1.00	0.29
*MRPL15*	NM_014175	2.13	0.44
*PCNA*	NM_002592	0.72	0.53
*ATP6V1C1*	NM_001695	1.13	0.53
*SCYL2*	NM_017988	2.13	0.57
*RRM2*	BE966236	0.72	0.57
*DLG7*	NM_014750	1.13	0.57
*KLHL12*	AK024412	0.91	0.59
*UBE2J1*	NM_016021	0.72	0.62
*SLMO2*	BF032213	0.68	0.62
*MLF1IP*	NM_024629	1.13	0.62
*FAM33A*	BE048371	0.72	0.62
*CCDC117*	AW070234	0.81	0.62
*TMTC3*	AA428286	0.55	0.72
*EIF3J*	BC002719	0.40	0.75
*TOP2A*	AU159942	0.33	0.76
*WASL*	AL523820	0.55	0.81
*FIGNL1*	NM_022116	0.55	0.81
*ABCD3*	BC009712	0.13	0.94

Genes were grouped according to the significance of them being enriched in endothelial cells compared with non-endothelial cells based on expression sequenced tags (ESTs). To account for multiple testing errors, an FDR was employed to calculate the significance of the genes being endothelial enriched (q<0.05). Bold text indicates genes that are significantly enriched in endothelial cells with a q-value of <0.05.

### Immunohistochemistry for LCM

Immunochemistry staining of sections for LCM was optimised for maintenance of RNA quality. For endothelial-specific CD31 staining, sections were fixed in acetone (5 min, −20°C), and incubated with primary antibody anti-CD31 (WM59, BD Pharmingen™) 1∶5 for 5 min followed by two PBS (Ambion) washes, biotinylated secondary antibody (Vectastain Universal Quick kit PK8800 – Vector labs) for 5 min, two PBS washes, StreptABCcomplex/HRP (Vectastain Universal Quick kit PK8800 – Vector labs) for 5 min, two PBS washes, amino-ethyl-carbazole (AEC, Dako K3464) for 5 min, two water washes (Ambion), Cresyl Violet 1∶20 in water (Ambion, AM1935) for 30 sec, two water washes and finally air-dried for up to 1 min with a hair-dryer. All antibody steps and AEC contained the RNase inhibitor 0.4 U/µL RNase Protector (Roche; Indianapolis, IN), and all PBS and water were nuclease free. All steps were carried out in RNase-free conditions. LCM was carried out immediately using the Zeiss Axiovert 135 with PALM Microbeam 3 System and RoboSoftware (Carl Zeiss Europe). In combination with CD31 expression, blood vessels were selected by morphology, where only small vessels with lumens or branching structures were collected. This method allowed for the isolation of whole blood vessel sections that include both endothelial cells and supporting cells. Approximately 200 blood vessels were captured onto adhesive lids of eppendorfs (PALM) per slide and lysed in 50 µl RNA lysis buffer (Qiagen RNeasy Micro Kit). Up to 3 hr of LCM was spent per slide. Blood vessels from up to 20 slides per patient were captured for combined RNA extraction. Details for staining for CD68 and CD31 are in Methods S1.

**Figure 5:**
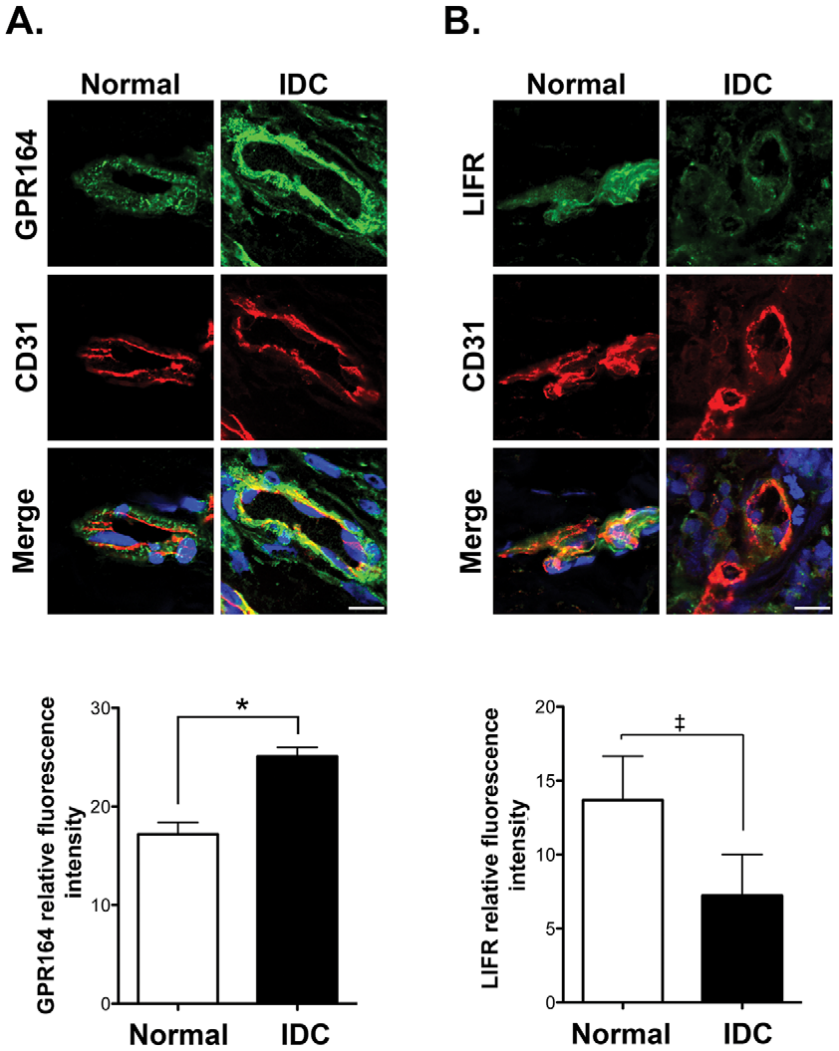
Validation of GPR164 and LIFR microarray expression data by immunofluorescence staining of human normal breast and IDC. Representative confocal images of vessels from normal and IDC breast sections immunostained for CD31 and either GPR164 (**A**) or LIFR (**B**). Relative expression levels were quantified and means + s.e.m. are given for each antigen. n = 6 individual tissue samples, *p<0.05, ‡ p<0.06, scale bars 50 µm.

### RNA extraction, reverse transcriptase and real-time PCR

RNA extraction was carried out using Qiagen RNeasy Mini or Micro Kit following the manufacturer's instructions with DNase treatment (Qiagen). RNA concentration and quality was analysed with Agilent 2100 Bio-analyzer (Agilent Technologies, Palo Alto, CA) using Agilent Pico kit or Nano kit and assessed using an Agilent software algorithm that allows the calculation of RIN with a numbering system from 1 to 10, with 1 being the most degraded and 10 being the most intact [32]. Only samples that had high RIN number of above 6 were considered for analysis. cDNA from RNA was synthesised using the High Capacity cDNA Archive Kit (Applied Biosystems, Foster City, CA) following the manufacturer's instructions. To determine if LCM captured samples had blood vessels, real-time PCR was used to quantify the expression of CD31 mRNA. Real-time PCR reactions were performed using the Applied Biosystem StepOnePlus^TM^ (Applied Biosystems, Foster City, CA) with SYBR green (Invitrogen). Primers against *CD31* 5′-AGAAAACCACTGCAGAGTACCAG-3′ forward and 5′-GGCCTCTTTCTTGTCCAGTGT-3′ reverse (Invitrogen, Paisley, UK). Glucose-6-phosphate dehydrogenase (*G6PD*) was used as a reference gene using primers 5′-AACAGAGTGAGCCCTTCTTAA-3′ forward and 5′-GGAGGCTGCATCATCGTACT-3′ reverse. Additional primers for validating differentially expressed genes are in supporting information (**Methods S1 and Table S1**).

**Figure 6:**
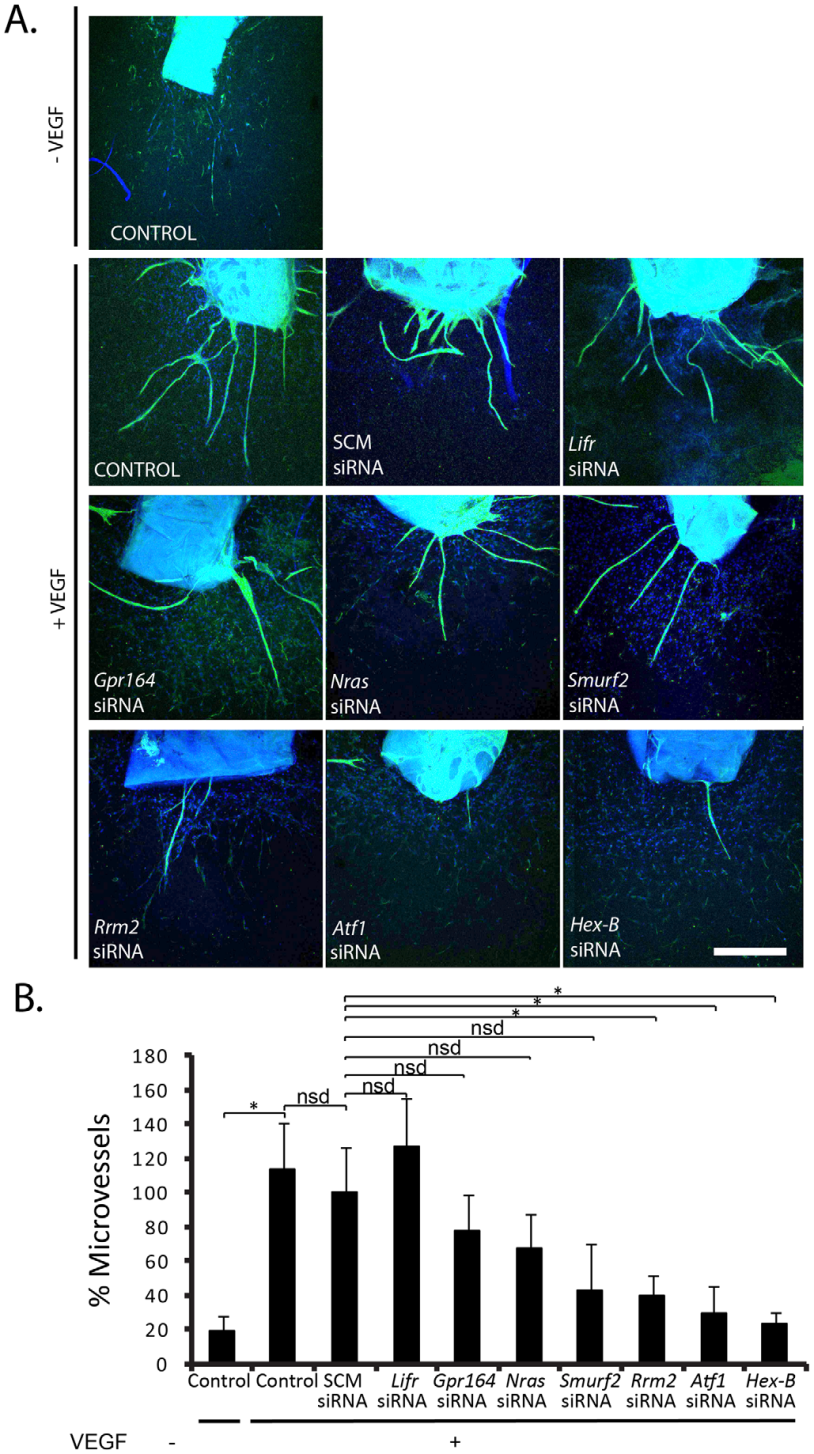
Depletion of Rrm2, HexB and Atf1 inhibits VEGF-stimulated angiogenesis. (**A**) Confocal micrographs of aortic ring microvessel sprouting immunostained with BS1-lectin (green) and DAPI (blue) following growth in serum free media or 30 ng/ml VEGF and treatment with siRNA targeting scrambled control (SCM), *Lifr, Gpr164, Nras, Smurf2, Rrm2, Atf1*, and *Hex-B*. (**B**) Bar-chart represents mean number of microvessel sprouts/aortic ring given relative to SCM with VEGF + s.e.m. *p<0.05, nsd, no significant difference. n = 12–24 aortic rings per test. Scale bar, 200 µm.

### Microarray experiments

RNA from LCM samples was amplified using the WT (whole transcriptome)-Ovation Pico RNA Amplification system (NuGEN) with a 2-cycle amplification following the manufacturer's instructions, and cDNA was fragmented and labelled using the FL-Ovation cDNA Biotin Module V2 kit. Although 2-cycle amplification may introduce a bias over 1-cycle, we were careful to control for this by amplifying both the cancer and normal samples identically. Labelled cDNA Microarray hybridisations were performed on HG-U133 Plus 2 arrays (Affymetrix) and gene expression data was analysed using Bioconductor 2.2 [33] running on R2.7.1. [34]. Normalised probeset expression measures were calculated using the ‘Affy’ package's Robust Multichip Average (RMA) default method. Differential gene expression was assessed between replicate groups using an empirical Bayes t-test as implemented in the ‘limma’ package [35]. The resultant p-values were adjusted for multiple testing using the False Discovery Rate (FDR) Benjamini and Hochberg method [36], where any probe sets that exhibited an adjusted p-value FDR q<0.05 were called differentially expressed. Two-dimensional hierarchical clustering of expression data using differentially expressed genes was performed using a Pearson correlation distance matrix and average linkage clustering [34]. All data have been deposited in a public database. Affymetrix data was also analysed with Ingenuity Pathways Analysis software (Ingenuity® Systems, www.ingenuity.com). Additional Microarray analysis was carried on human U87 xenograft samples (**Methods S1**).

### Endothelial specific genes

All genes found to be differentially expressed from the microarray results were compared to genes selectively expressed in endothelial cells from cDNA and SAGE library analysis. Briefly, libraries were divided into two pools; pool 1 contained endothelial cell libraries and pool 2 libraries of non-endothelial normal primary cell isolates. *In-silico* subtraction, employing maximum likelihood statistics, between both pools found endothelial-enriched genes and these results were used to prioritise LCM candidate genes. See Herbert *et* *al*. 2008 for a full description of methods [37]. The results shown are the FDR-adjusted q-values [38].

### Immunohistochemistry for GPR164 and LIFR

Frozen sections (8 µm thickness) were fixed in acetone for 5 min (−20°C), washed in PBS, blocked with 5% BSA/PBS for 30 min and incubated with primary antibodies (LIFR: 1/50, Santa Cruz sc-659; GPR164: 1/400, Abcam ab65759, CD31: 1/100, BD Pharmingen 550389) overnight at 4°C. Sections were washed in PBS before adding the appropriate fluorescent secondary antibodies for 1 hr at room temperature and mounted with ProLong Gold® anti-fade reagent with DAPI (Invitrogen P-36931). Sections were imaged using a LSM 510 inverted confocal laser-scanning microscope (Carl Zeiss Ltd., UK). For each channel, the detector gain and amplifier offset were set to display the full range of signal intensities within and between samples and then adjusted to exclude background. These settings were kept the same when imaging all the patients' samples. Consecutive sections from each patient were also stained with appropriate IgG control antibodies and imaged using the same settings. Staining was quantified in the CD31-positive blood vessels using ImageJ software. Blood vessels were identified by their CD31 positivity and the corresponding GPR164 or LIFR fluorescence intensity per pixel was measured. ImageJ software was used to evaluate the mean relative intensity of fluorescence for these markers in blood vessels within the sections. In order to normalise blood vessel-specific immunostaining for GPR164 and LIFR between samples, it was necessary to correct for differences in non-specific staining. This was done by analysing the mean fluorescence intensity of IgG non-specific staining in the CD31-positive blood vessels and then subtracting from the specific staining obtained with GPR164 or LIFR for each sample. This allowed us to overcome the heterogeneity of the non-specific staining between different patients. Four to five blood vessels from 6 normal breast and 6 breast IDC samples were quantified. Results are shown as the mean relative intensities of the samples ± s.e.m. Statistical significance was determined by Student's t-test at a level of p<0.05.

### Ex vivo *Aortic Ring Assays*

Thoracic aortas were isolated from adult C57BL6 mice and prepared for culture as described previously [39]. Depletion of target genes with siRNA was carried out essentially as described in Reynolds *et* *al*., [40]. Microvessels growth on aortic rings were quantified between 6–10 days. After maximum sprouting capacity was achieved, aortic rings were fixed and stained with BS1-lectin as described in Reynolds *et al*., [41].

## Results

### Laser Capture Microdissection of blood vessels from human breast tissue

Twenty-eight human IDC samples and 13 normal human breast samples were obtained and snap-frozen in liquid nitrogen. Although using snap-frozen tissue probably provides the best preservation of RNA without fixation, there are many factors, such as handling time and especially the interval between surgery and snap-freezing the sample, that can affect the quality of the RNA in breast tissue. Out of all the samples collected, only 16 IDC samples and 6 normal breast samples had high enough RNA quality (a RIN above 6) for further analysis according to Agilent lab-on-a-chip pKit bioanalyser (**Figure** **1A and B**).

An anti-CD31 antibody was used to identify the blood vessels in normal and breast IDC sections. This was in preference to other markers, such as CD146, since CD146, in addition to its expression in the vascular compartment, is expressed on a subset of epithelial cells in malignant breast [42]. Lastly, comparison of PECAM staining directly with CD146 staining has shown that, CD146 identifies a substantially higher number of non–blood vessels structures in serial sections of breast cancer [43]. Thus the use of PECAM as a marker of endothelial cells, together with blood vessel selection according to morphology, likely provides us with a highly specific method to detect blood vessels in breast cancer. The quality of the immunodetection was excellent for both breast IDC and normal sections and blood vessels were identified easily for LCM (**Figure** **2** **A and B**). By using a combination of both CD31-positivity and morphology, to select blood vessels with a lumen or a branched structure, we avoided the possibility of selecting myeloid cells that are CD31/CD68 double positive. (**Methods S1** **and Figure S1**).

Although good quality RNA was obtained from LCM samples (**Figure** **2C**), up to 70% of total RNA was lost following immunostaining compared with unstained equal sized tissue samples. Thus, using this staining protocol, 7 out of 16 cancer and 5 out of 6 normal samples gave sufficiently good RNA quality and quantity for Affymetrix analysis (**Methods S1** **and Figure S2**). Using real-time PCR we confirmed that the tissue samples isolated by LCM were enriched significantly for *CD31* when compared with whole control unstained sections (p<0.05, **Figure** **2D**).

### Identifying genes that are differentially regulated in blood vessels associated with human breast cancer

Using the WT (whole transcriptome) Ovation Pico RNA Amplification system, we amplified RNA levels to 2.6–6.3 µg of labelled cDNA from 5 IDC and 4 normal breast samples. The yield from the remainder of the samples was too low for utilisation. For Affymetrix gene expression analysis we compared laser-captured CD31-positive blood vessels from 5 IDC samples (2.8–6.3 µg cDNA) with vessels from 4 normal breast samples (2.6−3.9 µg cDNA). Other studies using frozen tissues gave an average present call rate of 26% [44]. We obtained present call rates of up to 59.2% from our samples, suggesting a good level of hybridisation in our experiments. Hierarchal clustering revealed that 3 IDC and 3 normal breast associated blood vessel samples correlated well and clustered into normal and cancer groups (**Figure** **3A**). Samples with low percent call rates corresponded to outliers that were omitted from the subsequent analysis (**Figure** **3A**). In total, 73 probe sets representing 70 genes were differentially expressed between normal and breast IDC associated blood vessels (**Figure** **3B**). Seven genes were downregulated (more than 10 fold) and 63 genes upregulated (5–72 fold) with FDR q<0.05 (**Tables** **1** **and** **2**). Using Ingenuity Pathway Analysis, we found that the 70 differentially expressed genes in human breast IDC associated blood vessels have molecular, cellular and signalling functions many of which are involved in angiogenesis (**Tables** **3** **and** **4**). Thus the upregulated gene list provided us with a cohort of potentially anti-angiogenic targets.

We then compared the list of 63 genes that were upregulated in IDC-associated blood vessels with a list of mouse stromal genes that were downregulated following treatment with the anti-VEGF agent (Bevacizumab) (For details on methods relevant to this section see Supplementary Information, **Method S1** **and Figure S3**). This allowed the identification of IDC-associated blood vessel genes that were likely to be regulated by VEGF. Bevacizumab is an anti-angiogenic agent that prevents human VEGF from binding and activating VEGF-receptor 2 on endothelial cells, resulting in the downregulation of stromal genes that contribute to angiogenesis. We injected BALB/c SCID mice with human U87 tumour cells and then treated the mice with Bevacizumab or placebo. Whole tumours, including tumour stroma, were then analysed using mouse-specific arrays. This approach enabled us to examine the effect of Bevacizumab on downregulating genes in the mouse tumour stroma. By cross-referencing the IDC blood vessel gene list with the genes downregulated after Bevazicumab treatment, we show that the greatest proportion (65%) of the genes upregulated in IDC blood vessels are also downregulated in the tumour stroma after Bevacizumab treatment. The results from this combined approach indicate that we have identified a group of genes that may also play an important part in VEGF-mediated angiogenesis. These data thus provided us with a further suggestion of the potential angiogenic function of the genes that were upregulated in IDC-associated blood vessels.

### Identifying genes enriched in endothelial cells

Targeting endothelial cells, specifically, is one approach to reducing the potential side-effects of novel anti-angiogenic strategies. Thus, we asked whether any of the genes that were upregulated in IDC-associated blood vessels were also enriched in endothelial cells. Earlier work from Herbert *et* *al*. found endothelial cell enriched genes using cDNA libraries [37]. By cross-referencing these genes with IDC-associated vessel genes, we found 6 genes that were expressed significantly in endothelial cells (q<0.05) compared with non-endothelial cells (**Figure** **4**). **Table** **5** shows the list of endothelial-expressed genes where *SMURF2, LIFR, HEX-B, C1orf107, ATF1* and *NRAS* were enriched significantly in endothelial cells (q<0.05). Together, these results indicate that by using this approach we can identify not only genes that are upregulated significantly in blood vessels associated with human breast IDC, but also further refine the list to mark those with an enriched expression profile in endothelial cells.

### Immunofluorescence validation of GPR164 and LIFR

The object of this study was to discover novel functional regulators of angiogenesis. Since mRNA levels do not always correspond to either protein levels or functionality, they may have limited physiological relevance. Thus we examined protein expression levels and functionality of a subset of candidate molecules identified in the arrays. In addition to the list of endothelial-enriched genes and those that were also downregulated by Bevacizumab, for validation purposes, we analysed the expression of proteins that are encoded by differentially regulated IDC genes that have no known roles in angiogenesis but were expressed at the cell surface. For example, GPR164 and LIFR are both found at the cell surface. By immunofluorescence microscopy we showed that GPR164 and LIFR were differentially expressed in breast IDC compared to normal breast samples, as our differential gene expression arrays predicted (**Figure** **5**). Immunofluorescence for other candidate proteins was not technically possible due to the lack of antibody reactivity in whole tissue sections.

### Identifying novel anti-angiogenic targets

Given that our goal, was to identify targets with anti-angiogenic potential we focused on siRNA-depletion of these candidate genes in aortic ring assays. We examined the angiogenic capacity of a subset of the 63 genes that were upregulated significantly in IDC-associated blood vessels. This subset of genes were selected on either their known cellular functions and/or enriched expression in endothelial cells. The selected genes included *NRAS, SMURF2, ATF1* and *HEX-B* because of their high expression in endothelial cells. In addition, given that *LIFR* was downregulated in IDC-associated blood vessels this gene was also included as a potential negative regulator of angiogenesis. *GPR164* was selected because it is expressed at the cell surface, making it potentially targetable, and has been shown to be upregulated in prostate cancer [45]. *RRM2* was selected because this molecule has been shown to be involved in cell proliferation and associated with VEGF production in cancer cells, but never studied in endothelial cells [46]. siRNA depletion of these genes in aortic ring assays was used to determine their role in inhibiting VEGF-induced angiogenesis. The degree of knockdown was confirmed by real-time PCR (**Methods S1** **and Figure S4**). Aortic rings were treated with siRNA for 24 hr, and cultured in 30 ng/ml VEGF or PBS as a control for up to 10 days (**Figure** **6**). Aortic rings responded to VEGF-stimulation with a significant (p<0.05) increase in microvessel number compared with PBS treated aortic rings (**Figure** **6B**). Treatment with siRNA against *Rrm2, Atf1* and *Hex-B*, reduced VEGF-stimulated microvessel sprouting significantly when compared with SCM siRNA treated aortic rings (p<0.05). No significant decrease in microvessel sprouting was observed with siRNA against *Lifr, Gpr164, Nras* or *Smurf2*. These results indicate that *Rrm2, Atf1* and *Hex-B* are positively involved in VEGF induced angiogenesis. Thus, our results establish novel roles for these molecules in VEGF-induced angiogenesis. Together these results suggest that, RRM2, ATF1 and HEX-B may be good candidates for anti-angiogenic therapy.

## Discussion

The VEGF-signalling blocker Bevacizumab, can provide overall survival (OS) benefit in colorectal, renal and some breast cancer patients [47]–[50]. Unfortunately however, not all anti-angiogenic approaches have been as successful and differential tumour responses, even for Bevacizumab, may reflect differential blood vessel molecular profiles from varying tumour types [5]–[9]. For example, phage-display peptide libraries have demonstrated heterogeneity in tumour-associated blood vessels [11]–[14]. In addition, blood vessels from ovarian cancer, lung cancer and melanoma have been reported to express significantly different vascular molecular profiles [5]. Importantly, results between studies have not always identified the same molecular profiles for tumour associated blood vessels possibly reflecting different methodological approaches. However, if we accept that different tumours have different vascular molecular profiles, we hypothesise that effective anti-angiogenic treatments will likely rely on tailoring new anti-angiogenic drugs to specific cancer vascular profiles.

Previously, the pit-falls in identifying specific cancer-associated vascular profiles have been related to methodology: expression analyses were done either on cultured cells or on whole cancer samples. The problem being that these systems do not discriminate blood vessels from the rest of the cancer and can also introduce molecular changes associated with culturing cells [6]–[8]. In addition, knowing which blood vessel marker to use has been an issue. For example, selection of CD146-positive cells from colorectal cancer [8] was used to identify blood vessel. However CD146 is also expressed in smooth muscle cells, which are abundant in this cancer type, suggesting that any differential gene expression data would include both myofibroblast and blood vessel genes. Here, we have overcome such problems by using laser capture microdissection of CD31-positive blood vessels to isolate blood vessels without altering, drastically, their molecular make-up. Although previous work has demonstrated that LCD of factor VIII-positive blood vessels identifies differentially regulated genes associated with breast cancer, none of these genes are proven to have anti-angiogenic efficacy. This is likely because factor VIII is not a marker of angiogenic, but rather quiescent, blood vessels and is also found in adjacent extracellular matrix [9], [30], [51]–[54]. In addition, we have used the 2-cycle amplification Nugene Ovation kit to amplify RNA, which is preferable to the T7Oligo(dT) method used by Bhati *et* *al*. [9], [55] because it has the advantage of amplifying higher numbers of genes from small amounts of RNA [55]. Variations in the differentially expressed genes identified by us or other groups [5], [9] likely reflect differences in either the cancer types studied, or the method used.

We have identified 70 differentially regulated genes that are associated with CD31-positive blood vessels in human breast IDC when compared with normal breast (5 fold or more, q<0.05). These genes potentially encode biomarkers and/or anti-angiogenic targets relevant to breast cancer. Given that vascular maturity is defined as vessels with a defined basement membrane and endothelial cells with close cell-cell junctions and association with pericytes/smooth muscle cells [56], LCM captured vessels may also contain some supporting cells and our data may highlight genes associated with vascular maturity. This would be of interest to pursue since vascular maturity has been associated with resistance to anti-VEGF therapy [57].

By comparing our differentiated gene list with genes that were regulated by inhibiting VEGF with Bevacizumab in human glioblastoma U87 xenografts, we found that 65% that were highly expressed in IDC blood vessels were also downregulated by Bevacizumab. A glioblastoma cell line was chosen, in these xenograft experiments, because it is highly vascularised, reproducible, does not have a high basal necrosis and responds well to Bevacizumab. Glioblastoma is also a tumour type for which Bevacizumab is approved. Since hypoxic cancer cells are the major source of VEGF, the effect of Bevacizumab, which is specific to human VEGF, was selected. Although comparison of our data with EST libraries may be limited by the exclusion of any genes that are enriched only in supporting cells, our result suggests that the genes that were found to be highly expressed in breast IDC associated vessels may also play an important part in VEGF-mediated angiogenesis.

To inhibit angiogenesis it may be important to target endothelial cells specifically. By comparing the list of 63 genes that are upregulated significantly in IDC associated blood vessels with genes found to be enriched in endothelial cell ESTs, six were significantly expressed in endothelial cells compared with non-endothelial cells. Indeed we have shown that out of the endothelial enriched genes, *Smurf2, Hex-B, Atf1*, and *Nras*, plus *Rrm2*, which has known roles in tumour biology, that depletion of *Rrm2, Atf1* and *Hex-B* have anti-angiogenic consequences in *ex vivo* mouse aortic ring assays. These data validated our approach in the discovery of novel anti-angiogenic targets.

The *RRM2* gene encodes a ribonucleotide reductase small subunit of ribonucleotide reductase enzyme (RNR) [58] and plays an essential role in DNA synthesis, repair and cellular proliferation [59]. A role for RRM2 in blood vessels has not been documented, but it can enhance angiogenesis by upregulating VEGF in oropharyngeal carcinoma cells [46]. The downregulation of RRM2 by Bevacizumab, suggests that it is VEGF-regulated. Together our data, with previous studies, suggest that inhibiting RRM2 may have anti-angiogenic and anti-tumour effects.

*HEX-B* (hexosaminidase subunit beta) is involved in catalysing the degradation of the ganglioside GM2 [60]. The role for HEX-B in angiogenesis has not been explored previously, but ganglioside can modulate cell signalling. Gangliosides shed by tumour cells can also enhance VEGF-induced angiogenesis [61]–[64]. In addition, recent studies have indicated that interferon tau (IFNT) treatment of cycling ewes increases the endometrial expression of HEXB and that this was associated with increased Hif-1α levels [65]. Bevacizumab treatment is also known to increase hypoxia in the tumour environment and Hif-1α expression [66]. Thus it is temping to speculate that this regulates the increased HEXB in our Bevacizumab experiments. How HEX-B in endothelial cells regulates angiogenesis will need further investigation.

*ATF1* regulates downstream target genes involved in growth and survival [67]–[69]. In endothelial cells ATF1, when phosphorylated, has been shown to upregulate COX2 [70]. Since VEGF also upregulates COX2, and this correlates with increased tube formation [71], we speculate that high expression of ATF1 in IDC blood vessels may enhance endothelial cell responses to VEGF. Indeed our data corroborate this, since depletion of *ATF1* can reduce VEGF-mediated angiogenesis.

In summary, we have successfully combined CD31 immunostaining and LCM to analyse gene expression in breast cancer associated blood vessels. Although beyond the scope of this study, future work will establish the biomarker value of the 63 genes that are upregulated in blood vessels associated with human breast cancer. Moreover, since siRNA depletion of *Hex-B, Atf1* and *Rrm2* inhibit VEGF-stimulated angiogenesis, our approach has demonstrated novel anti-angiogenic targets. Future work will be required to investigate their *in vivo* roles.

## Supporting Information

Figure S1 **CD31-positive blood vessels are negative for the myeloid marker CD68 in human breast cancer.** For laser capture microscopy we identified blood vessels by their expression of CD31 (*red*) and their morphology *i*.e., structures with a clear lumen and or branched morphology. However, CD31 has also been shown to be expressed in some myeloid cells. Here we demonstrate that CD31 structures, with a clear lumen and or branched morphology are CD68 (*green*) negative, a biomarker for myeloid cells. Our results suggest that CD31-LCM captured blood vessels from breast samples were myeloid negative.
(TIF)

Figure S2 **RNA profile of the six LCM captured samples used for gene expression array.** (**A**) RNA gel-like profile and (**B**) histograms of RNA samples analysed with an Agilent bioanalyser. The 28S and 18s distinctive ribosomal RNA bands were observed in all 6 samples, and RIN ranged from 6–9.1.
(TIF)

Figure S3 **Transcripts in the upregulated IDC vessel signature that respond to anti-VEGF treatment.** Comparison of upregulated laser capture blood vessel human IDC genes with those differentially expressed in the mouse tumour stroma following Bevacizumab treatment. U87 xenograft bearing mice were treated, or not, with Bevacizumab and stromal gene profiles were compared with the human IDC blood vessel gene signature. C1–C5, controls; BEV1-BEV4, Bevacizumab-treated tumour stromal samples. Out of the 51 genes that were upregulated in human IDC blood vessels, 41 genes were down regulated following Bevacizumab treatment indicating their possible involvement in VEGF-stimulated angiogenesis. Colour scale indicated genes that were upregulated (red) or down-regulated (blue) in the endothelial cell signature. The heat-map is standardised per gene.
(TIF)

Figure S4 **Gene expression following siRNA treatment.** (**A**) Primary endothelial cells and (**B**) aortic rings were transfected with indicated siRNA and mRNA expression was measured by real-time PCR. Scrambled (SCM) siRNA was used as a negative control. Gene expression is given as a ratio to *Actin* mRNA expression, as an internal control, and the data in the graph is presented as fold-change relative to control sample. p<0.05, n = 3.
(TIF)

Methods S1 **Methods and Materials used for supplementary data.**
(DOC)

Table S1 **List of genes and their primer sequences used for validating differentially expressed genes in primary mouse endothelial cells with qPCR.**
(DOCX)
