# Supplementary figures and images for: Gene Expression Analysis in Human Breast Cancer Associated Blood Vessels
Source: PLoS One. 2012 Oct 2;7(10):e44294. doi: 10.1371/journal.pone.0044294 (PMC3462779; doi:10.1371/journal.pone.0044294)

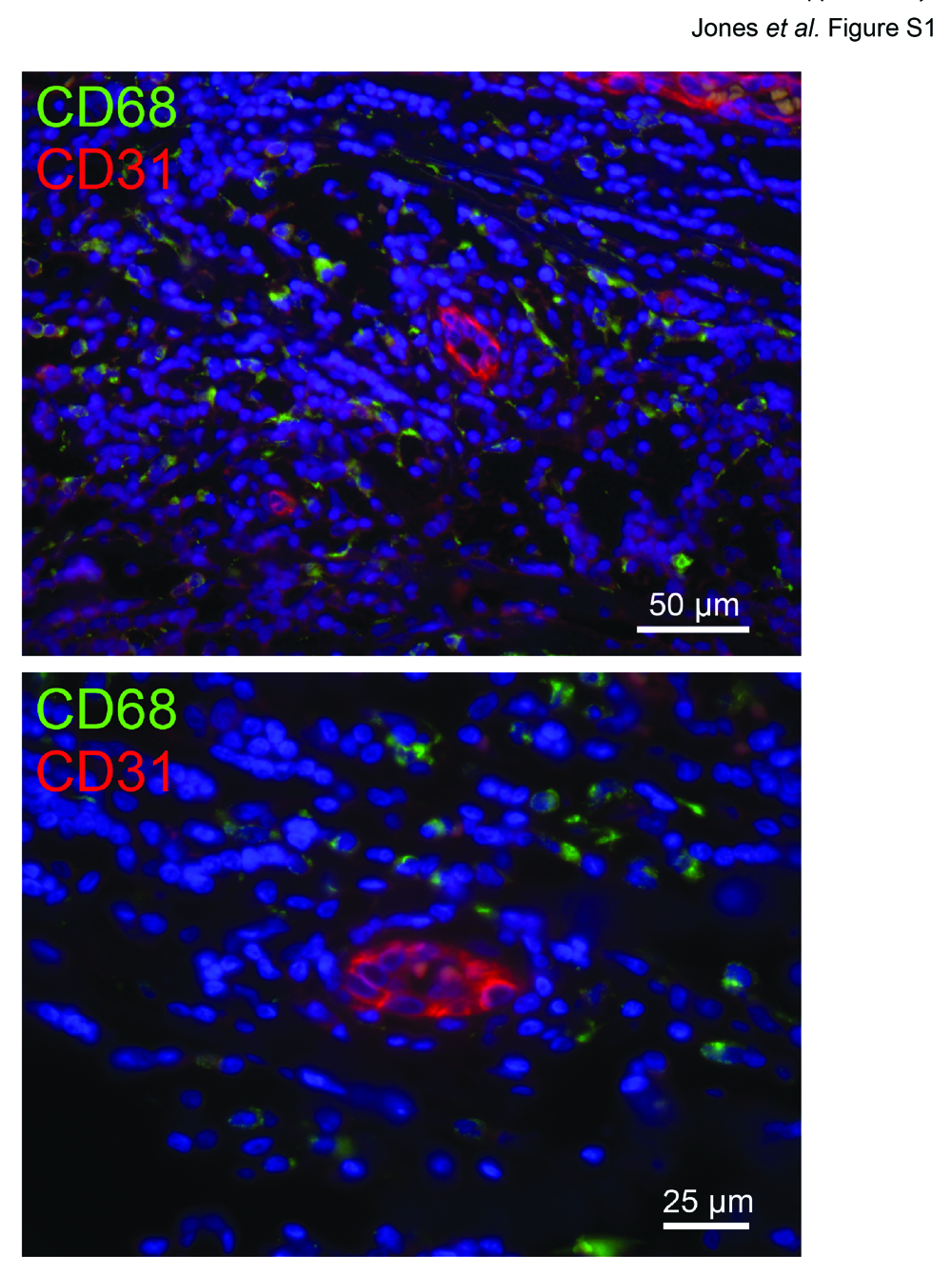

Supplement: Figure S1 — CD31-positive blood vessels are negative for the myeloid marker CD68 in human breast cancer. For laser capture microscopy we identified blood vessels by their expression of CD31 (red) and their morphology i.e., structures with a clear lumen and or branched morphology. However, CD31 has also been shown to be expressed in some myeloid cells. Here we demonstrate that CD31 structures, with a clear lumen and or branched morphology are CD68 (green) negative, a biomarker for myeloid cells. Our results suggest that CD31-LCM captured blood vessels from breast samples were myeloid negative. (TIF) [file pone.0044294.s001.tif]

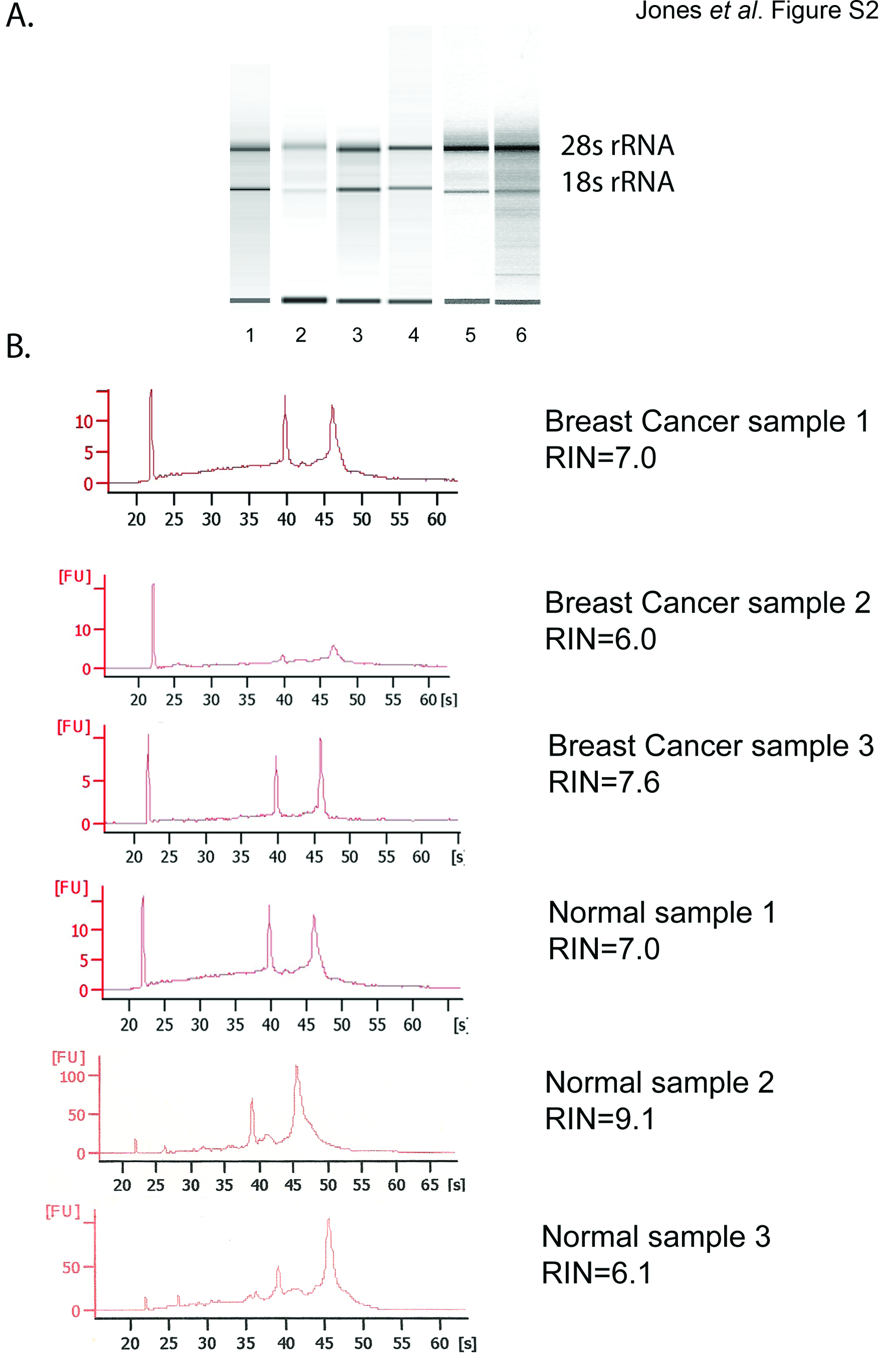

Supplement: Figure S2 — RNA profile of the six LCM captured samples used for gene expression array. (A) RNA gel-like profile and (B) histograms of RNA samples analysed with an Agilent bioanalyser. The 28S and 18s distinctive ribosomal RNA bands were observed in all 6 samples, and RIN ranged from 6–9.1. (TIF) [file pone.0044294.s002.tif]

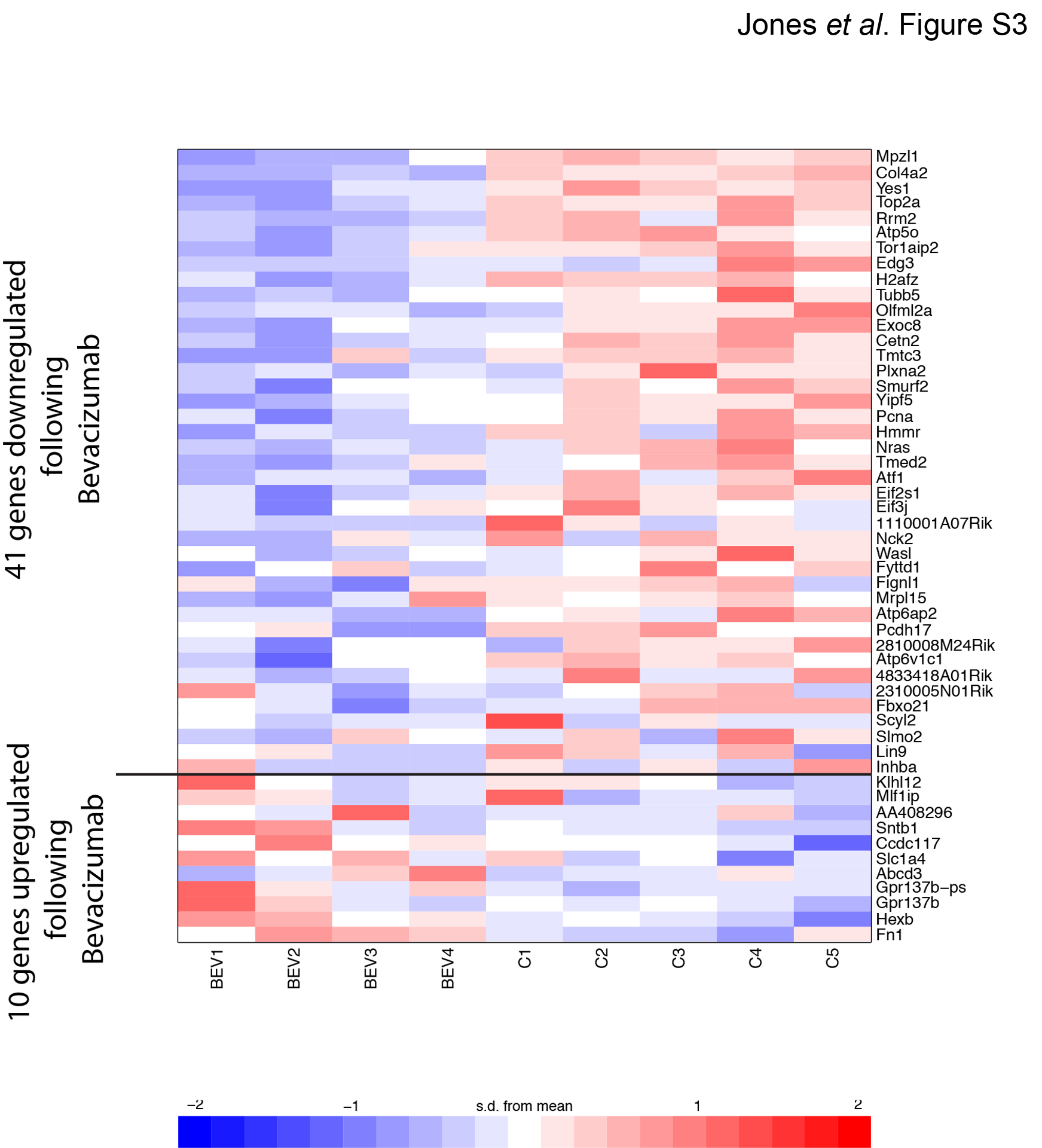

Supplement: Figure S3 — Transcripts in the upregulated IDC vessel signature that respond to anti-VEGF treatment. Comparison of upregulated laser capture blood vessel human IDC genes with those differentially expressed in the mouse tumour stroma following Bevacizumab treatment. U87 xenograft bearing mice were treated, or not, with Bevacizumab and stromal gene profiles were compared with the human IDC blood vessel gene signature. C1–C5, controls; BEV1-BEV4, Bevacizumab-treated tumour stromal samples. Out of the 51 genes that were upregulated in human IDC blood vessels, 41 genes were down regulated following Bevacizumab treatment indicating their possible involvement in VEGF-stimulated angiogenesis. Colour scale indicated genes that were upregulated (red) or down-regulated (blue) in the endothelial cell signature. The heat-map is standardised per gene. (TIF) [file pone.0044294.s003.tif]

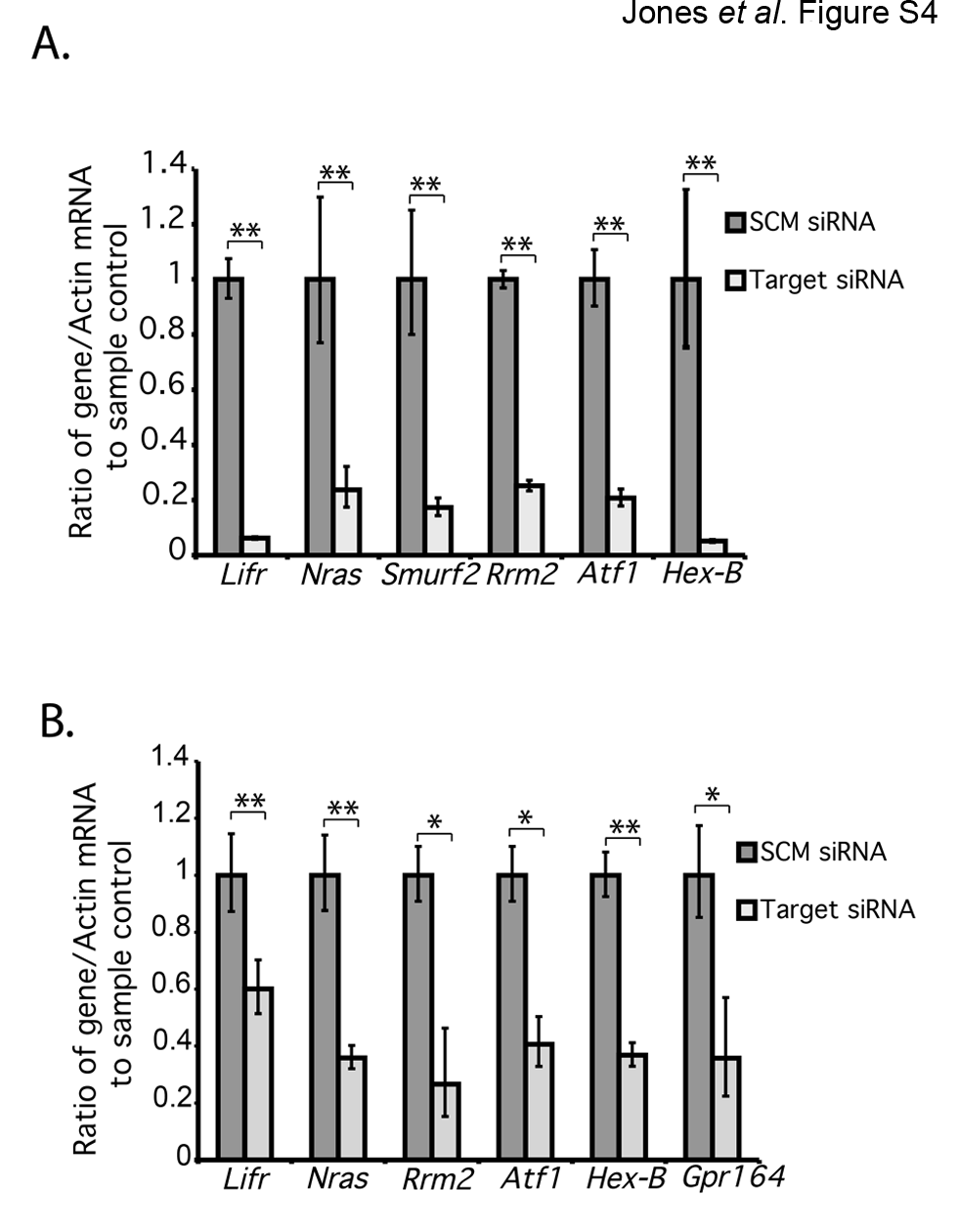

Supplement: Figure S4 — Gene expression following siRNA treatment. (A) Primary endothelial cells and (B) aortic rings were transfected with indicated siRNA and mRNA expression was measured by real-time PCR. Scrambled (SCM) siRNA was used as a negative control. Gene expression is given as a ratio to Actin mRNA expression, as an internal control, and the data in the graph is presented as fold-change relative to control sample. p<0.05, n = 3. (TIF) [file pone.0044294.s004.tif]
