# Supplementary material for: Gene Expression Analysis in Human Breast Cancer Associated Blood Vessels
Source: PLoS One. 2012 Oct 2;7(10):e44294. doi: 10.1371/journal.pone.0044294 (PMC3462779; doi:10.1371/journal.pone.0044294)
Supplement: Methods S1 — Methods and Materials used for supplementary data. (DOC) [file pone.0044294.s005.doc]

**Methods S1**

**CD68 and CD31 staining of breast sections**

Eight μm breast sections were de-waxed and rehydrated in alcohol gradient and followed by citrate-buffer antigen retrieval. Sections were washed in PBS and blocked for 1hr in 10% NGS. Sections were immunostained with anti-CD68 (mouse, Dako M0814) at 1/100 and anti-CD31 (rabbit, Abcam ab76533) at 1/100 in 1% NGS overnight at 4^o^C. Sections were washed in PBS and incubated with secondary antibody anti-mouse Alexa-488 (1/100) and anti-rabbit Alexa-546 (1/100) in 1% NGS 2hr at RT. Sections were washed and mounted with ProLong Gold® anti-fade reagent with DAPI (Invitrogen P-36931). Sections were imaged using a LSM 510 inverted confocal laser-scanning microscope (Zeiss). Vessels expressing CD31 and CD68 were quantified (n=102).

**Primary lung endothelial cell isolation**

Lung endothelial cells were prepared as described previously from wild-type C57BL6 mice . Lungs were minced; digested with 0.1% collagenase type I (Gibco Invitrogen, Ltd, Paisley, UK) in PBS for 30 min; passed through a 70 μm-pore cell strainer (BD Falcon, Bedford, MA, USA); resuspended in MLEC media containing 50:50 mix of Ham’s F-12:DMEM medium supplemented with 20% FCS, 20 μg/ml endothelial mitogen (Biogenesis, Poole, UK), 1 μg/ml heparin, glutamine and antibiotics; and plated onto tissue-culture flasks pre-coated with a mixture of PureCol^TM^ (Inamed, Fremont, CA, USA), human plasma fibronectin (Calbiochem, Beeston, UK), and 0.1% gelatine. Endothelial cells were cultured and purified over a 2 to 3-week period by a series of magnetic immunosorting including a single negative sort using antibodies to the Fc III /II receptor, to remove macrophages, followed by at least two positive sorts using antibodies to ICAM-2, to enrich for endothelial cells.

**siRNA transfection**

For primary MLEC, 24hr prior to transfection, endothelial cells were counted and seeded at 6x10^4^ cells per well of a 6 well-plate, in medium without antibiotics, to generate a 30-50% confluent monolayer on the day of transfection. For mRNA knockdown an ON-TARGETplus SMARTpool (Dharmacon) was used. This pool consists of 4 individual siRNAs, each targeting a different region of the same mRNA. For control, scrambled siGENOME non-targeting siRNA pool (SCM) was used (Dharmacon). For aortic rings treated with siRNA, 1mm-thick rings were transfected in 1 ml of Opti-MEM^TM^ medium with the indicated siRNA “smart” pool duplexes (final concentration of 100 nM) using Oligofectamine^TM^ (Invitrogen). Transfections were performed in 24-well plates with ≤24 rings/well. After 48hr aortic ring were removed, and RNA was extracted.

**RNA extraction**

RNA was extracted from the cells using RNeasy Mini Kit (Qiagen) following manufacturer’s instructions. The quantity and the quality of RNA was assessed using NanoDrop ND 1000 Spectrophotometer (NanoDrop Technologies, Wilmington, DE) and the Agilent 2100 Bio-analyzer (Agilent Technologies, Palo Alto, CA) respectively.

**Reverse transcriptase and quantitative PCR**

cDNA was synthesized by reverse transcribing RNA using the High Capacity cDNA Archive Kit (Applied Biosystems, Foster City, CA) following manufacturer’s instructions. Real-time quantitative PCR (qPCR) reactions were performed using 2-5ng of cDNA with 50-300nM primers in triplicate with SYBR green (Applied Biosystem StepOnePlusTM) following manufacturer’s instructions. Conditions for the PCR reaction were 2 min at 50°C, 10 min at 95°C and then 40 cycles, each consisting of 15 sec at 95°C, and 1 min at 60°C. **See table S1** for list of primers and sequence (all primers were supplied by Invitrogen, Paisley, UK). Relative quantitation of gene expression was performed using comparative Ct method. All calculations are based on the mean value of PCR reactions performed in triplicate.

**Xenograft tumour model plus Bevacizumab**

All protocols were carried out under home office regulations. The human glioblastoma U87-EV (10^7^) were subcutaneously implanted as 100 μl cell suspension with an equal volume of Matrigel (BD Bioscience) into 7 to 8-week-old female BALB/c SCID mice (Harlan Sprague Dawley, Inc., Indiana) . Each group consisted of 5 mice. Animal welfare was monitored daily and tumour growth was measured using callipers. Tumour size was calculated from a formula (V=LxWxHxπ/6). When tumour reached 0.35-0.45cm^3^, the mice were treated by intraperitoneal injection of either Bevacizumab (10mg/kg body weight) or same amount of PBS as a control. After 3 days, a second dose of Bevacizumab was given. Four hours after the second dose of treatment, the mice were sacrificed and the tumours were collected. Half of each tumour sample was freshly frozen in liquid nitrogen for RNA extraction. The RNA was used for microarray analysis.

**Microarray analysis after Bevacizumab treatment**

Microarray hybridization for the human U87 xenograft samples were preformed on mouse 430 2.0 Affymetrix arrays to assess mouse stromal responses. Data was GCRMA processed, quantile normalized and logged base2 (http://www.bioconductor.org). Matching between mouse and human Affymetrix Chips was done using Biomart (http://www.biomart.org). When two mouse probesets were matched to the same human gene, the probeset with the larger absolute effect was selected.

**Reference**

1. Reynolds LE, Hodivala-Dilke KM (2006) Primary mouse endothelial cell culture for assays of angiogenesis. Methods Mol Med 120: 503-509.

2. Li JL, Sainson RC, Shi W, Leek R, Harrington LS, et al. (2007) Delta-like 4 Notch ligand regulates tumor angiogenesis, improves tumor vascular function, and promotes tumor growth in vivo. Cancer Res 67: 11244-11253.
