# Supplementary material for: Gene Expression Analysis in Human Breast Cancer Associated Blood Vessels
Source: PLoS One. 2012 Oct 2;7(10):e44294. doi: 10.1371/journal.pone.0044294 (PMC3462779; doi:10.1371/journal.pone.0044294)
Supplement: Table S1 — List of genes and their primer sequences used for validating differentially expressed genes in primary mouse endothelial cells with qPCR. (DOCX) [file pone.0044294.s006.docx]

**Table S1. *List of genes and their primer sequences used for validating differentially expressed genes in primary mouse endothelial cells with qPCR.***

| Gene | Forward | Reverse |
| --- | --- | --- |
| *Actin* | aaggccaaccgtgaaaagat | gtggtacgaccagaggcatac |
| *Smurf2* | Tcgtggagaagaaggtcttga | gtgacaggagatacaaccattcc |
| *Atf1* | aagaagagaaatacgactgatgaaaaa | ttttccagaacagcaacacg |
| *Nras* | ttgagacctcagccaagacc | tggcgtatctcccttaccag |
| *HexB* | tttggcaagaagtttttgatga | ttccacacttcgactactgtgc |
| *Rrm2* | tttctttgcagcgagtgatg | cgggcctctgtaacttgaac |
| *Gpr164* mouse homolog-*Olfr558* | GCAGCCTGCCAGACCTCCCT | GCCCACCATACTGAAGGAATCAGGA |
| *Lifr* | aaagctaattccaagaaagaagtga | gcaacatggtaagttgaatcctc |
